# On the Road to End Pig Pain: Knowledge and Attitudes of Brazilian Citizens Regarding Castration

**DOI:** 10.3390/ani10101826

**Published:** 2020-10-08

**Authors:** Maria José Hötzel, Maria Cristina Yunes, Bianca Vandresen, Rita Albernaz-Gonçalves, Raphaela E. Woodroffe

**Affiliations:** 1 Laboratório de Etologia Aplicada e Bem-Estar Animal, Universidade Federal de Santa Catarina, Rod. Admar Gonzaga 1346, Itacorubi, Florianópolis 88034-001, Brazil; mcyunes@hotmail.com (M.C.Y.); vandresen.bianca@gmail.com (B.V.); rita.albernaz@santarosa.ifc.edu.br (R.A.-G.); raphaela.w@hotmail.com (R.E.W.); 2 Instituto Federal Catarinense, Campus Santa Rosa do Sul, Santa Rosa do Sul, SC 88965-000, Brazil

**Keywords:** animal welfare, boar taint, immunocastration, public perceptions, risk

## Abstract

**Simple Summary:**

We conducted two surveys to explore the attitudes of Brazilians (*n* = 1209) towards different piglet castration methods (surgical castration without or with pain control and immunocastration) and the production of entire males. Qualitative analyses indicated that moral objection towards inflicting pain and suffering to pigs was the main reason for the greater support for all the alternatives compared to surgical castration without pain control. Yet, support for all alternatives was somewhat limited by concerns with reduced meat quality, which some participants associated with boar taint or other residues in meat. Moving away from surgical castration without pain control is a necessary step for the pig industry to retain its’ social license.

**Abstract:**

We explored the attitudes of Brazilians towards different methods to deal with boar taint in pork (surgical castration without pain control, SC; surgical castration with pain control, SC+PC; immunocastration, IC; raising entire males, EM). Two surveys (Sv1, *n* = 441 and Sv2, *n* = 768) containing closed and open questions were conducted. Nearly 70% of Sv1 and Sv2 participants were unaware that meat of entire males may have boar taint and that SC is widely used in pig production in Brazil. In Sv1, acceptability of SC+PC (63%) and IC (53%) was greater than of SC (15%). In Sv2, acceptability of IC (55%) and EM (52%) was greater than of SC (18%). Open-ended responses indicated that participants objected to inflicting pain to pigs to attain a production goal, and were concerned with organoleptic traits and risks of exogenous residues in pork. Participants’ views regarding the potential increases in the cost of meat due to adoption of alternative methods varied; some argued that avoiding pain justifies an increase in the price of pork and others that this would impact especially lower income citizens. Our findings indicate that participants opposed surgical castration without pain control, and supported alternative methods. However, the concern with potential risks of presence of residues in meat, expressed by a few participants, may need to be addressed among consumers.

## 1. Introduction

Castration of piglets is a routine practice in pig farms around the world. The main reason for this procedure is to eliminate boar taint, an unpleasant flavor caused by androstenone and skatole, two substances that accumulate in fat tissue and release an unpleasant smell and taste when pork is cooked [1,2]. The most commonly used method is surgical castration of neonatal piglets [3,4,5]. Surgical castration without analgesia induces endocrine and behavioral responses that are considered indicators of pain; for example, newly castrated pigs spend less time nursing than non-castrated pigs, remain inactive longer while awake, and show more pain-related behaviors, such as prostration, stiffness, and tremors [6].

The use of pain control protocols may address the pain and suffering. However, any method used must be suitable in the farm context; i.e., it must be easy to perform, not require expensive equipment, and promote significant reduction or elimination of pain, discomfort, and stress for piglets. Studies validating the effectiveness of different pain control protocols have reported mixed results [7,8,9,10,11]. Furthermore, handling and recovery associated with anesthesia and analgesia are a source of stress, and drugs used may have temporary nociceptive effects [6,12]. In Europe, pain control is increasingly used, but still mostly restricted to countries where its use is legally required [3].

An option to avoid surgical castration is immunocastration. This method consists of an injectable vaccine that immunizes the pig against its endogenous GnRH, which inhibits testicular development and function, reducing skatole and steroids levels in the blood [13,14]. The method has positive effects on productivity and meat quality [15,16], and produces pork with acceptable organoleptic qualities [17]. Immunocastration avoids the labor involved on surgical castration but adds the need for two injections, one of them when the pigs are older and heavier; also, concern with work-related accidents requires trained staff [18]. The production of entire males with acceptable levels of boar taint is also feasible through a combination of early slaughter and specific feeding and environmental measures [19]. Raising immunocastrated or entire males may offer economic benefits such as faster growth, enhanced feed conversion, and leaner carcasses. However, entire animals need to be slaughtered before puberty to avoid boar taint, which results in lighter carcasses [18,19,20]. Additionally, control of sexual and aggressive behavior in entire males requires adequate management of the environment [19]. Genetic selection of animals with low levels of skatole and aldosterone, or gene edition to produce animals with these traits may be possible, but both require further development and have additional implications on fertility [21,22].

Cultural and regulatory issues may influence farmers’ attitudes towards the alternative measures to control boar taint and influence adoption. Many farmers consider methods to replace surgical castration without pain control unnecessary, ineffective, costly, or impractical [23,24,25]. In some countries, the use of pharmacological tools requires the involvement of a veterinarian, which acts as a barrier to the adoption of pain mitigation [26,27]. These factors are reflected in regional differences in the adoption of the methods. European countries successfully raise entire male pigs [3], while immunocastration is increasingly used in countries like Brazil, New Zealand, and Australia, but not in Europe and the United States, where stakeholders are concerned with consumers’ acceptability of immunocastration [17,20].

However, adopting animal management practices that resonate with societal values is a key element for the sustainability of the livestock industry. Together with environmental, social, and food security issues, animal welfare is cited as a main challenge facing agriculture today [28,29]. In order to maintain its’ social license, the livestock industry must regard the values of citizens and consumers. Although citizens in general are unaware of the husbandry practices and technologies used in animal production, when asked about surgical castration of pigs without anesthesia, most classify these practices as detrimental to animal welfare [30,31,32,33]. Housing and management practices that are highly prevalent in intensive pig farming such as confinement, feeding practices that leave animals hungry for most of the day, and painful mutilations have become highly contentious [34,35]. In 2002, the World Organization for Animal Health (OIE) passed a resolution to develop international animal welfare standards, which Brazil embraced, launching a series of measures and legislation aimed at regulating animal production [36]. Attentive to their public image, food retail companies are pushing their suppliers to transition to better animal husbandry systems, such as housing pregnant sows in groups and ending piglets’ surgical castration and other painful mutilations, e.g., [37]. Brazil is the world’s fourth largest pig producer, after China, the European Union, and the United States; although the country is the fourth largest exporter after the European Union, United States, and Canada, 80% of the pig meat produced in Brazil is consumed in the country [38]. Despite the importance of agriculture for the Brazilian economy, little is known about the attitudes of its citizens towards the production systems [36]. Although surgical castration without pain control is widely used in Brazil, a growing proportion of the farmers are moving to immunocastration [39], with support from large retailers and industry stakeholders (see [37,40]). One study showed that Brazilian citizens had low awareness about pig castration but, when informed about it, expressed negative attitudes due to the pain it causes to the animals [33]. However, there is no information about the opinion of the public regarding the acceptability of immunocastration or raising entire males. 

This study comprised two surveys, aiming to explore Brazilian citizens’ attitudes towards different methods to deal with the problem of boar taint in pork: surgical castration with or without pain control, immunocastration, or raising entire males. A secondary aim of the first survey was to explore the attitudes towards potential increases in pork price resulting from changing castration practices.

## 2. Materials and Methods 

This study was approved by Ethics Committee on Experimentation of the Santa Catarina State University, PP 2.229.017 and 3.495.015. The two surveys will be thereafter called Sv1 and Sv2.

Until August 2020, slaughter of uncastrated male pigs was prohibited in Brazil, and for this reason, the option “raising entire males” was not included in the first survey. After extensive discussions and consultation with stakeholders, this prohibition was lifted in August 2020 [41]. To present a complete overview of citizens’ attitudes to the issue of castration of pigs, a second survey was carried out, including the option of raising entire males for pork production. However, given the health regulations posed by the COVID-19 pandemic, it was not possible to carry out face-to-face recruiting, the method of recruitment used in 2017. Thus, participants for the second survey were recruited online. 

### 2.1. Recruitment and Questionnaires

Conditions to participate in the research were that the participant was a Brazilian citizen and at least 18 years old. The identity of the participants was not requested to guarantee their anonymity. Participant recruitment for Sv1 took place during October and November of 2017, at the Hercílio Luz International Airport (Florianópolis, Brazil). The airport location was chosen because of the intense movement of people [42]. People waiting in the public airport hall located before security were approached and invited to participate in the study. Participants were asked if they would be willing to take a survey about animal production, with no specification of the nature of the issue, to reduce self-selection bias. Participants received a consent form and were asked to read and sign before taking the survey. The researcher stayed visible to answer any doubt, but did not provide any extra information while the participant filled out the questionnaire. Permanent residence in one of the three southern states of Brazil (i.e., Rio Grande do Sul, Santa Catarina, and Paraná states) was a condition to participate in Sv1. Participants for Sv2 were recruited online between June and August 2020. An advertisement was posted on Brazilians’ social media inviting the public to participate in a survey about animals, without further information on the survey subject. The first question asked participants to identify themselves in relation to animal husbandry for food production (‘I am totally opposed to raising animals to produce food,’ ‘I support the rearing of animals for food production, provided it is done in an ethical manner’, or ‘I support the rearing of animals for food production without restrictions’), and only those that stated support for farm animal production were retained for this study. 

In both surveys, participants were invited to read a short text before answering the questions (Box A1), which outlined reasons behind castration and described castration techniques. In Sv1, participants were informed that Brazilian legislation states that all slaughtered male pigs must be castrated. In 2020, the legislation was revised removing this prohibition [41]. Thus, in Sv2, this information was not included and a short text about raising entire males was added. Detailed description of the questionnaires is available on Supplementary Materials Tables S1 and S2. 

Briefly, the questionnaire used for Sv1 was 6 pages long and included a total of 37 closed questions and 1 open question. After reading the text, participants were randomly assigned to rate and justify the acceptability of one of the three different techniques described there: “surgical castration” (SC), “surgical castration using medication to control pain” (SC+PC), or “immunocastration” (IC). If the participant answered about castration with pain control or immunocastration, two extra questions were asked—the acceptability if the cost to produce increased up to 10%, and up to 30% of the current price. This was followed by an open question asking to justify their answers. Participants were then asked to rate their acceptability of the two other methods. 

The questionnaire used for Sv2 included a total of 27 closed questions and 1 open question. Participants started the survey indicating which of the following elements they considered to be the most important in the production of meat for human consumption: avoiding techniques and forms of rearing that cause pain to the animals; avoiding management practices that deprive animals of freedom of movement; or ensuring meat production without undesirable residues. Participants were then randomly assigned to answer about “surgical castration”, “immunocastration”, or “raising entire males” (EM), indicating on a 5-point Likert scale, whether they considered the treatment appropriate, whether they approved it, and whether they considered it acceptable. This was followed by an open question asking to justify their answers. 

At the end of both questionnaires, participants were asked if they were aware of some pig production practices common in Brazil before participating in the survey (the order of the statements was randomized). Next, there were questions addressing participants’ socio-demographic information relating to sex, age, education, geographic region of current residence, urban or rural area, involvement with agriculture, and monthly family income (only in Sv2). The last two questions in Sv2 asked participants, “Do you believe that the current context associated with COVID-19 may have influenced your responses?”, with answers yes/no, followed by “please, justify your answer” and if they had or had ever had a castrated dog. 

### 2.2. Analyses

#### 2.2.1. Quantitative Analyses

Data of the two surveys were analyzed separately, using R language (R CoreTeam, 2016). In Sv1, a total of 441 valid usable questionnaires were collected and transcribed to the file for analysis. Participants that left closed questions unanswered and those that wrongly answered an attention question (‘Select “false” to validate your answers’) were excluded (*n* = 63). Comparisons of Likert scale ratings between two groups was done with the Wilcoxon rank-sum test (V value reported), and effects of demographic variables on acceptability were analyzed using the Kruskal-Wallis rank test (χ2 value reported). 

In Sv2, a total of 1223 responses were collected. Participants who identified themselves as totally opposed to livestock production (*n* = 222) and those who had some involvement with livestock production (farmer, professional, or student) (*n* = 233) were excluded from the analysis, resulting in a sample of 768 responses: surgical castration (*n* = 261), immunocastration (*n* = 260), raising entire males (*n* = 247). Consistency of the three first attitude questions (i.e., how much they approved the method, how they considered it acceptable and adequate) was assessed using Cronbach’s alpha; as the alpha coefficient was >0.93, these responses were averaged to create a mean for each participant for their “attitude” towards the method (surgical castration, immunocastration, or raising entire males). The effects of each of the socio-demographic questions (sex, age, education, urban/rural, region of the country, and income), perceived influence of the COVID-19 pandemic on answers, if they were aware of boar taint and that piglets are castrated, ownership of a castrated dog, and the three methods (i.e., surgical castration without pain control, immunocastration, and raising entire males) on attitudes were tested using generalized linear models. Post-hoc comparisons were performed using a Tukey HSD test. 

For the descriptive analysis of acceptability of methods in both surveys, Likert responses to the question on acceptability were considered. Likert 1 and 2 were grouped as ‘unacceptable’ and Likert 4 and 5 were grouped as ‘acceptable’, Likert 3 as ‘neutral/does not know’.

#### 2.2.2. Qualitative Analyses

Open responses were submitted to thematic analysis [43]. This method allows identifying and interpreting patterns across the data and involves careful reading and rereading of the text for the development of codes and key themes. Thematic analyses of Sv1 and Sv2, respectively, were done by BV/MJH and RAG/MJH. In both cases, the pair ran the analyses together, comparing their results and discussing any discrepancies and ambiguities until agreement was reached. When responses bridged more than one theme, they were coded into multiple themes. In Sv1 and Sv2, respectively, responses from 233 and 565 participants were used, as not all participants answered or gave a meaningful answer. Quotes that were representative of specific themes that appear in the paper were translated from Portuguese to English by MJH and revised by MCY and BV. 

## 3. Results

### 3.1. Quantitative Findings

Demographic data of both surveys are shown in Table A1. Participants’ distribution of sex, age, and place of residency approximately corresponded to the Brazilian population of southern Brazil in Sv1 and with the Brazilian population in Sv2 [44]. However, in both surveys, a higher proportion of participants had undergraduate education. Participants’ income, only asked in Sv2, was well distributed in the sample, but a greater proportion of people had higher incomes than the general Brazilian population. In Sv1 and Sv2, respectively, 26% and 31% participants had grown up with some contact with farm animal production, but were not involved with agriculture. 

Participants considered meat eating very important (Sv1 = 18%; Sv2 = 25%), important (Sv1 = 39%; Sv2 = 23%), intermediate (Sv1 = 23%; Sv2 = 29%), not important (Sv1 = 13%; Sv2 = 14%), and not important at all (Sv1 = 7%; Sv2 = 9%) (χ2 = 39, 4 df, *p* < 0.0001). Frequencies of meat consumption (beef, pork, chicken) were: 5–7 times a week (Sv1 = 44%; Sv2 = 52%), 3–4 times a week (Sv1 = 29%; Sv2 = 27%), up to 2 times a week (Sv1 = 20%; Sv2 = 16%) (χ2 = 6.3, 2 df, *p* = 0.04). Only 7% in Sv1 and 4% in Sv2 never ate any meat (similar to the 7% of the Brazilian population, [45]). In addition, 18% participants in Sv1 and 10% in Sv2 did not consume pork. Reasons for eating pork were organoleptic qualities of the product (Sv1 = 51%; Sv2 = 50%; χ2 = 3, 1 df, *p* = 0.08), tradition (Sv1 = 29%; Sv2 = 34%; χ2 = 1.7, 1 df, *p* = 0.2), and price (Sv1 = 27%; Sv2 = 47%; χ2 = 36.6, 1 df, *p* < 0.001); only few participants marked the options impact on animal welfare (Sv1 not asked; Sv2 = 8%) and impact on the environment (Sv1 not asked; Sv2 = 8%). 

**Figure 1:**
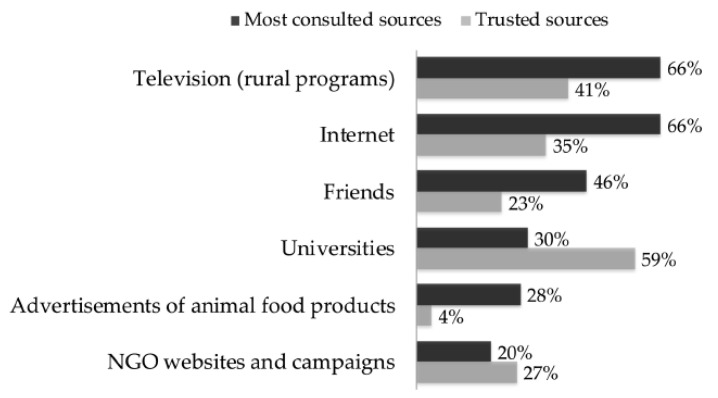
Sources of information on animal related issues (Sv1, *n* = 441). Black bars = most consulted sources, grey bars = trusted sources.

In Sv1, the sources participants consulted for information on animal production were different from the sources they trusted (Figure 1). 

In Sv2, when asked which of three options was the most important element in the context of production of meat for human consumption, 71% of participants said avoiding husbandry practices that cause pain to animals; 15% avoiding husbandry practices that deprive animals of freedom of movement; and 14% ensuring that meat is produced free of residues. Approximately half (54%) of the Sv2 participants had or had had a castrated dog. Only 10.3% of the participants responded yes to the question “Do you believe that the current context associated with COVID-19 may have influenced your responses?”, and of the few that justified the answer expressed concern about confinement of animals (*n* = 5), zoonoses (*n* = 4), and food consumption habits that may be related to zoonoses (*n* = 3).

#### 3.1.1. Attitudes towards the Different Alternatives to Deal with Boar Taint in Pig Meat

Table 1 summarizes descriptive data on acceptability of the different alternatives presented to participants in both surveys. All alternative methods had greater acceptability than surgical castration without pain control, which was considered unacceptable by over two thirds of the participants, both in Sv1 and Sv2. Immunocastration also had similar acceptability in both surveys.

**Figure 2:**
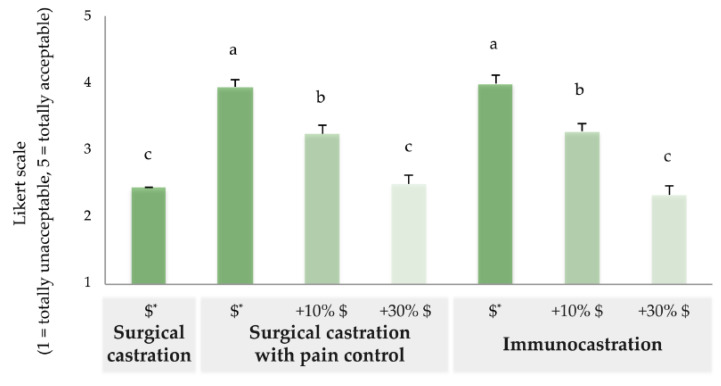
Acceptability of piglet castration methods and influence of change in price (Sv1, n = 441. $* = price not mentioned; +10%$ and +30%$ = increase compared to current price). Different letters indicate *p* < 0.05.

In Sv1, acceptability of surgical castration with pain control was higher, followed by immunocastration and surgical castration (SC vs. SC+PC, V = 43211, *p* < 0.0001; SC vs. IC, *V* = 5801, *p* < 0.0001; SC+PC vs. IC, *V* = 11955, *p* < 0.002). Acceptability of SC+PC was higher among male participants (χ2 = 13.6, 1 df, *p* = 0.0002), and tended to be higher for SC (χ2 = 4.6, 1 df, *p* = 0.034), but was not influenced by participant’s sex for IC (χ2 = 0.4, 1 df, *p* = 0.5). Age, education, and growing up in an agriculture environment did not influence acceptability. Acceptability tended to be higher among participants that consumed meat 5 to 7 days a week compared to those that ate meat rarely or never (SC, χ2 = 9.9, 4 df, *p* = 0.042; SC+PC, χ2 = 15.9, 4 df, *p* = 0.003; IC, χ2 = 11.8, 4 df, *p* = 0.018). Attribution of importance to meat consumption influenced acceptability of SC (χ2 = 13.6, 4 df, *p* = 0.008), but not of SC+PC (χ2 = 7.0, 4 df, *p* = 0.13) or IC (χ2 = 9.1, 4 df, *p* = 0.06). When presented with a scenario of 10% or 30% increase in price of pork compared to current price, the acceptability of SC+PC and IC changed (χ2 = 156, 6 df, *p* < 0.001). Descriptive data are shown in Figure 2. 

In Sv2, attitudes were influenced by the method of dealing with boar taint (surgical castration without pain control = 2.08; immunocastration = 3.38; entire male 3.28; SEM 0.08; *p* = 0.001) and sex of participant (female = 2.72; male = 3.11; SEM 0.06; *p* = 0.001). Interactions between methods and sex were tested and excluded from the model, as they were not significant (*p* > 0.2). Attitudes towards immunocastration and entire males did not differ (*p* = 0.74) and both were higher than towards surgical castration without pain control (*p* = 0.001).

#### 3.1.2. Awareness of Pig Production Practices

Participants’ awareness of pig production practices in Brazil was in general low among participants in both surveys (Table 2). Sv1 also included three questions about antibiotics used for animal health; 63% of the participants said that before taking the survey, they were aware that “antibiotics used in animal production can contribute to the resistance of microorganisms to antibiotics”; 60% that “antibiotics fight only bacteria (not viruses and parasites)”; 54% that “several antibiotics used in animal production are also used in human health”. In addition, 19% answered yes to the question “have you or someone in your family or close friends experienced a medical condition involving antibiotic resistance”.

### 3.2. Qualitative Findings

The thematic analyses of the responses identified six themes common to both surveys, and one theme that only emerged in Sv1 (impact of cost of meat for consumers, which could be associated with the question regarding price meat, only asked in that survey). The main theme identified in both surveys was “pigs’ pain and suffering” (Table 3). Data were calculated dividing the number of references to a given theme by the number of participants. Many sentences bridged more than one theme and were thus coded into multiple themes. A selection of representative quotes that validate the analysis described below and give a rich overview of participants’ attitudes is displayed in the Appendix C (Table A2). 

#### 3.2.1. Survey 1

Reducing pain and suffering in animals subjected to castration was the main justification for support of immunocastration or the use of pain mitigation, as well as for rejection of surgical castration without pain control. Participants argued that pigs should be castrated by the method that results in less pain and suffering for the animals, because it is ethical, results in better quality of the final product, or both. A few participants expressed concerns that immunocastration or the anesthetics and analgesics used to control pain could leave residues in the meat, which would pose health risks to humans. 

In this survey, possibly because we investigated the implications of a potential increase in the price of the final product with the adoption of the alternative castration methods on attitudes, this was the second most prevalent theme. While 58% of those that mentioned the issue reported concerns that the lower income population would have reduced access to meat in the face of increases in cost, 41% commented that an increase in meat price would be acceptable or even necessary to reduce pigs’ pain and suffering. Some participants discussed farm animal welfare as a public good, arguing that producers and consumers should not be responsible for paying this price increase and that the government should bear this cost. Others mentioned the need for legal measures, and government support and oversight to encourage the adoption of castration methods that cause less pain and suffering to animals. For some, information about the castration method on the labelling of products would be important for consumers to accept the price increase.

#### 3.2.2. Survey 2

Concerns with pigs’ pain and suffering, and arguments involving ethics and animal welfare were the two main justifications for support or rejection of methods proposed to deal with the problem of boar taint in pig meat. The pain and suffering of pigs, and ethical or animal welfare repercussions of surgical castration without pain control were the most discussed topics by participants that justified why they opposed this practice. Avoidance of pain and stress of surgical castration was the main justification for participants to support immunocastration or raising entire males. For some participants, the practice of surgical castration would be acceptable as long as it was done with pain control. Some argued that it is unethical that people involved in animal production cause pain to pigs to achieve production goals, given that they are the gatekeepers of these animals’ welfare and health. Others argued that consumers must adopt responsible meat consumption behaviors or demand that meat be produced without animal suffering. 

Some participants supported surgical castration in pigs because of their experience with castration in pet animals. For these participants, given that this procedure is commonplace and acceptable for dogs and cats, there would be no reason why it would not be acceptable for pigs. Likewise, they said that just as anesthetics and painkillers are used for dogs and cats, there would be no justification for not treating pigs in the same way.

In contrast, arguments to support castration without pain control were the facts that surgical castration without pain control is a consolidated practice in pig production and the most favorable option for the producer, or the belief that young pigs do not feel pain in castration or that it is short-lived.

A few participants showed concerns about production losses due to raising entire animals and the organoleptic characteristics of the meat. Some showed concerns about the risk of residues in the meat of immunocastrated pigs. Loss of naturalness—either due to castration or immunocastration—was mentioned by a few participants, who said that pigs should be reared more naturally, without using chemical products, and should not be subjected to procedures that alter their nature. 

## 4. Discussion

Despite some methodological differences between the two surveys used in this study, a common finding was the rejection of surgical castration without pain control by over two thirds of the participants and greater acceptability of alternative methods. The reasons provided by participants in the two surveys to justify these attitudes were ethical concerns related to pain and suffering in animals, while acceptability of the alternative methods was influenced by concerns with potential exogenous residues or boar taint reducing meat quality, as well as the impact of the methods on animal welfare, naturalness, or production aspects. Increasing the price of pork reduced acceptability of both immunocastration and use of pain control. However, participants’ views regarding possible impacts of changes to improve pig welfare on price of pork varied. For some, avoiding pain justifies an increase in meat price, but others argued that this would be unfair, especially for lower-income citizens.

Pain and suffering was the main reason underlying low acceptability of surgical castration without pain control and support for the alternative methods. Participants also cited avoiding pain as a main priority in farm animal production. Likewise, animal welfare experts in the UK identified poor pain management as the most important animal welfare concern for pigs [46]. A few participants argued that surgical castration was essential to guarantee productivity, a view most often shared by industry stakeholders, who consider some painful practices acceptable to maintain farm production systems [25,27]. Yet, other studies support that pain caused by human intervention is one of the most important public concerns regarding animal production systems. For example, when asked to describe an ideal pig farm model, citizens without ties to the pig industry said that pigs must live without pain and suffering [34]. European citizens strongly oppose surgical castration without pain control [47,48], which has led Europe to discuss steps to gradually ban the practice [3]. Our findings show that Brazilian citizens share these views, which should encourage the local industry to move to alternative methods. 

For many participants in this study, pain control would be a solution for the problem. However, on-farm adoption of pain relief protocols is limited by scientific and technical uncertainties regarding their effectiveness [7,8,9,10,11], farmers’ attitudes towards these tools [23,25], and legal restrictions to the use of these drugs by non-veterinarians [49]. Discussing the issue of castration from an ethical perspective, Palmer et al. [50] argued that, considering that the realistic alternative in pig production is surgical castration, immunocastration appears as the best immediate solution. This was the case for some participants in this study, who argued that immunocastration was the best alternative given that it is practical and effective and that raising entire males posed the risk of lower meat quality. 

As shown in surveys with European citizens [30,32,47] some participants considered immunocastration unacceptable based on the perception that the method is unnatural. Several studies have reported public concern about unnaturalness in pig production, as well as preference for systems with the least possible human interference [30,34,51]. Citizens and consumers often refer to hormones, antibiotics, pesticides, transgenic components, and feed additives collectively as “chemicals” and link them with lack of naturalness in production systems, and with lower animal welfare and product quality [35,51,52]. As shown in this study, Jansen et al. [53] found that some people have negative attitudes towards chemicals in food and consider them unnatural and detrimental to health, and associate “chemical” with danger. Concern that immunocastration (and to a lesser extent, pain medications) would leave residues in the meat was also reported by participants, as shown by others [47,54,55]. This was a reason stated by a few participants to support surgical castration without pain control. This concern may also explain why some participants explicitly expressed opposition to inflicting pain to animals, yet had negative attitudes towards castration with pain control or immunocastration. Although some studies indicated that consumers in Europe have positive attitudes towards immunocastration [31,32,56], a widely reported limitation for adoption of this method is producers’ and industry stakeholders’ concern regarding consumer acceptance [17,20,23,48]. Indeed, health and safety aspects are the animal production traits that consumers in different parts of the world perceive as most important when assessing alternatives to pig castration [30,33,48,57].

Low awareness of pig castration issues, identified in both surveys, has also been reported in a previous study in Brazil [33] and in several European countries [4,30,31,32,47,55,56,58]. We also identified low awareness of tail docking, adding to previous studies that found that Brazilians have low awareness of animal production practices in general [33,59,60]. Expecting such low levels of awareness, we provided participants some information on why and how pigs are castrated, aiming to help people to evaluate the alternative methods in the context of commercial pig farming. However, it has been shown that simply informing lay citizens of the technical reasons behind practices that they may perceive as detrimental to animal welfare does not necessarily lead to support [55,57,59,61]. Yet, reducing animal suffering may be a powerful argument for the public to accept technologies that they otherwise reject on the grounds of risk for humans [33,56,62,63]. 

To maintain public trust, it is essential that these technologies are satisfactorily proven safe and effectively reduce animal suffering, as these are important conditions for acceptance [33,62,63]. In the case of immunocastration, there is sufficient scientific knowledge demonstrating the absence of residues potentially harmful to humans (see [18,20]). Nonetheless, some participants in both surveys expressed distrust in immunocastration despite the explanatory text explicitly mentioning the contrary, which may have some explanations. For example, the name of a technology influences public attitudes [64], so the term immunocastration may have induced some people to think of controversies like vaccines or biotechnologies, adding to distrust. Additionally, it has been shown that interpreting risk messages is not straightforward, and lay understanding may not be in line with the intended scientific meaning [53]. Lastly, part of the participants said that they did not trust most of the sources of information on animal production, which may be why some participants implied having low confidence in the information presented. A previous survey with Brazilian lay citizens reported complaints about misinformation and discontentment with information sources on transgenic crops, which caused distrust [65]. Credibility and shared values with providers of information are two key factors influencing trust in information sources about new technologies [66]. As in other studies [55,67], television and Internet were the sources of information about farm animal production most accessed by the participants. These sources bring negative news [55,68] more often than information that may help citizens understand the basic elements of the discussion. Therefore, the public must be clearly informed, as by doing so trust is built, and trust is an important factor behind public acceptance [69]. 

Even though pork is cheaper compared to beef, more than half of the participants in both surveys said that price was not an attribute to choose pork. Our findings suggest that some Brazilians would consider price increases to avoid painful procedures in pigs acceptable. However, given that Sv1 participants were recruited at an airport, these opinions are restricted to middle and upper-class participants. As these participants recognized, the price of meat is more relevant for lower-income citizens. Some participants implied that they viewed animal welfare as a public good, as suggested in the study of Lagerkvist and Hess [70]. For these participants, pig welfare must be improved, but not at the expense of the consumer. The scenarios presented to participants, of 10 and 30% increase in the price of pork, are unrealistically high, especially given that immunocastration is used in Brazil, with no direct impact on the price of pork for consumers. Yet, participants did not challenge the veracity of the price increases proposed, which may be explained by the low awareness of pig production systems. It is possible that participants assessed the price increase proposed in the survey based on the price of organic products in the region [71]. Some Europeans may be willing to pay more for pig castration methods that take animal welfare into consideration [30,31,54,55,72]. In contrast, one study showed that Brazilians’ views on pig production systems did not influence pork purchasing choices [60]. The willingness to pay for meat produced in systems perceived as more “animal friendly” is poorly explored in Brazil. Considering that over 80% of the pork produced in Brazil is consumed in the domestic market [38], the issue warrants further investigation. Importantly, further studies must include the voice of lower income citizens, who may be more affected by increases in the price of meat resulting from animal welfare related changes in production systems and practices, as hinted by many participants. A fact to consider is that other changes in pig production practices that may impact on costs of production, like the transition of individual to group housing for gestation sows, are happening as a result of pressure from retailers, without direct consultation with consumers [36].

Animal welfare and environmental impacts of meat production were not listed by most participants as reasons to choose meat, despite the relevance of these issues for livestock production [28,29]. However, meat-eating habits had some influence on acceptability of surgical castration and its alternatives. As shown in a Chilean study [57], acceptability of surgical castration tended to be greater among participants that ate meat more frequently and those that considered meat more important. This finding is relevant because more than half of the participants in both samples rated eating meat as important, and over three quarters ate meat 3 to 7 days a week. These findings are consistent with a previous study in Brazil showing similar patterns [63], as well as with the average meat consumption of the Brazilian population, of 99.8 kg per person per year (13.7 kg pork), which makes it one of the ten countries with the highest meat consumption in the world [73]. Meat attachment and meat consumption habits are associated with moral disengagement regarding meat eating, i.e., the deactivation of moral self-regulation to reduce cognitive dissonance when considering the impacts of meat eating [74]. This helps explain the apparent contradiction between the concern with animal welfare expressed by participants and the low importance assigned to this trait as a criterion for choosing meat. In contrast, participants cited tradition and organoleptic qualities among the most important attribute to choose pork, as shown by others [31,32]. Altogether, these findings corroborate the conclusions of Macdiarmid et al. [75], that public opinion around eating meat is associated with personal, social, and cultural values, and suggests that meat eating habits influence attitudes towards farm animal issues. Attitudes towards husbandry practices like castration may become increasingly negative over time, given the trends towards lower meat consumption, even in Brazil [63]. 

Only few participants raised concerns that the practice of castration is a mutilation and therefore unnatural or cruel. It has been shown that acceptance of farm animal mutilations is low, but realizing an advantage from the practice and that it can be done in the absence of pain leads to greater support [58]. Participants discussed the two aspects (need for the procedure and pain) to justify their attitudes towards the different methods. More than half of the participants in Sv2 had a castrated dog, confirming our assumption that many people have personal experience with the practice in pet animals. To avoid introducing a bias, this was the last question in the Sv2, and as a consequence, few people covered the issue in the open-ended question. Even so, some participants’ responses suggest that experience with castration in pets may contribute to castration of animals being somewhat normalized. A survey in the UK found that citizens were predominantly supportive of dog and cat neutering [76]. Dog owners in The Netherlands said that correcting unwanted behaviors was an important reason to have their male dogs neutered [77], supporting that perception of necessity may lead to support of the practice. In another study, Spanish consumers that were not aware that pigs are castrated said that it would not be a welfare issue if pigs were castrated in the same way domestic pets [78], something that was also suggested by some participants in this study. In conclusion, experience with castration in pets may give people an opportunity to reflect about the pain and stress involved in the procedure, and may further contribute to their conclusion that causing pain to castrate animals is both unnecessary and avoidable. 

This study was based on two convenience samples, and the results should not be considered representative on a regional or national scale. Participants’ sex was approximately equally distributed between the two samples and the demographics of the Brazilian population. Both samples contain a greater proportion of well-educated citizens, which is likely linked with citizens’ wealth; although this is not likely to influence people’s values towards pain and suffering in animals, it may influence their perception of priority of animal welfare relative to other issues of food production. Participants in Sv2 were somewhat younger than in Sv1, and from all regions of the country, whereas Sv1 participants were from southern Brazil. Moreover, other limitations to the generalization of results of this study are the use of different recruitment methods (in person and online) and a 3-year gap between the two surveys, added to the unusual situation posed by the COVID-19 pandemic in 2020 (e.g., confinement in the cities that had lockdowns, unemployment, sickness in the families or social groups). Only a few of the participants in Sv2 recognized that the pandemic might have influenced their responses or their views, but this may be because the survey was carried out in the early days of the pandemic. 

The only demographic characteristic of participants that influenced attitudes towards piglets’ castration was sex, with women showing more negative attitudes towards surgical castration in both surveys, as shown in two previous studies [33,57], and adding to several others that have shown that women have greater concerns regarding farm animal welfare [52]. More than 25% of the participants in both samples reported growing up with some contact with farm animal production. In the online recruiting, it could be argued that this was caused by participants’ self-selection based-interest in the subject. However, Sv1 participants were randomly recruited in person in an airport, yielding similar result; additionally, in both cases, participants were not informed of the nature of the survey before they accepted to participate. This may in fact reflect the recent urbanization of Brazil, that changed from 45% people living in urban centers in 1960 to 85% in 2010 [79].

Our results, and previously published literature, indicate unequivocally that there is a need to end the practice of pig castration with methods that cause pain. It is important to note, though, that piglets’ castration is one procedure among a myriad of other production practices that challenge pig welfare [80,81]. To preserve its’ social license, the pig production sector needs to move beyond avoiding practices that cause pain and stress to animals, as current understanding is that farm animal welfare is more than just avoiding animal suffering [82]. Thus, ending painful castration methods needs to be allied to changes that address public concerns, such as intensive feeding and housing practices and other painful procedures [34,35,61,83]. Broader changes may be beneficial for pigs and also for production. For example, rearing pigs in lower stocking density and with environmental enrichment may be a requirement to raise pigs with tails and testicles [19,20,84] and would have further positive impact on pigs’ welfare [85]. A recent study showed that replacing surgical castration with immunocastration, avoiding tail docking, teeth clipping, and providing environmental enrichment to pigs resulted in lower physiological stress and risks for injuries and death, and improved growth with positive economic returns—but not when these changes were made individually [86]. Thus, changing one specific practice while maintaining others may incur in costs for producers without real benefits for the animals, and fail to meaningfully contribute to the social acceptability of the production system. 

## 5. Conclusions and Recommendations

Participants in the two surveys were opposed to piglets’ surgical castration without pain control. The main reason for this position was the perception that the practice causes unnecessary pain and stress to animals and is therefore ethically unacceptable. The three alternatives presented in the study—using pharmacological tools to control pain during and after castration, immunocastration, and raising entire males—had more support than surgical castration without pain control. Considering that all the alternatives to replace the procedure are successfully used on farms in different parts of the world, the pig industry has no justification for not acting proactively. The choice of method to replace surgical castration and still produce pork of acceptable quality must fulfil the societal value of not causing pain and suffering to animals, as well as consumers’ demand for safe and high-quality products and, additionally, consider environmental, economic, production, and practical implications of each option. These factors may vary according to local farming culture, as well as regulatory, industry, and market requirements. For two reasons, immunocastration is a suitable method to replace surgical castration in Brazil. Firstly, farmers are increasingly adopting it for at least a decade, with positive results; secondly, using pain medications would require overcoming current cultural and regulatory limitations. Raising entire males is a new option for the Brazilian industry, and thus farmers’ and consumers’ attitudes should be further investigated, as this study suggests that there may be a potential niche market for its products. In summary, our findings indicate that the Brazilian public supports and prefers pig castration methods that do not cause pain to pigs; they also suggest that the concern with potential risks of presence of residues in meat, expressed by a few participants, may need to be addressed among consumers.

## Figures and Tables

**Table 1:** Descriptive data on acceptability (%) of the different methods to deal with boar taint in pig meat.

Survey	Method	Acceptable	Unacceptable	Neutral/Does Not Know
	Surgical castration	15	73	12
Sv1 (*n* = 441)	Immunocastration	53	27	20
	Surgical castration with pain control	63	22	15
	Surgical castration	18	67	15
Sv2 (*n* = 768)	Immunocastration	56	25	19
	Entire male	52	32	16

**Table 2:** Awareness (%) of some pig production practices.

Question	Sv1 *n* = 441	Sv2 *n* = 768
Most pigs’ feeds used in Brazil are produced with transgenic soy and corn	69	68
In pig production in Brazil, antibiotics are commonly used to improve performance. Many are the same as those used in human health	42	41
Meat from non-castrated pigs slaughtered after puberty may present boar taint	20	34
Pig producers tail dock piglets to avoid them from being bitten by others in their group ^1^	26	17
Male pigs used in meat production undergo castration or immunocastration	30	33

^1^ Sv2 included the following information: “This is done on the same day of castration and without the use of anesthesia or analgesia”.

**Table 3:** Emerging themes (%) in the justification for attitudes towards different methods to deal with boar taint in pigs (Sv1, *n* = 441; Sv2, *n* = 768). SC = surgical castration without pain control; IC = immunocastration; SC+PC = surgical castration with pain control; EM = raising entire males.

		Castration Method				
	Survey	SC	IC	SC+PC	EM	Total
Pigs’ pain and suffering	Sv1	54	54	52	-	53
	Sv2	55	55	-	45	52
Ethical and animal welfare concerns	Sv1	27	7	34	-	24
	Sv2	33	12	-	16	20
Risks for consumers (residues in meat)	Sv1	11	16	9	-	12
	Sv2	1	11	-	3	5
Organoleptic traits of meat (boar taint)	Sv1	2	3	4	-	3
	Sv2	3	9	-	21	10
Effects on production	Sv1	11	1	2	-	5
	Sv2	3	8	-	12	7
Naturalness	Sv1	0	3	1	-	1
	Sv2	4	7	-	5	5
Impact of cost of meat for consumers	Sv1	5	61	39		33
	Sv2	-	-	-	-	-

## Supplementary Materials

The following are available online at https://www.mdpi.com/2076-2615/10/10/1826/s1, Table S1: Script of the questionnaire survey 1 with the specific questions of the study and demographics, Table S2: Script of the questionnaire survey 2 with the specific questions of the study and demographics.

## Appendix A

Box A1 Information text used in the questionnaires. **Sv1** “The majority of pigs in Brazil are slaughtered at around 6 months of age, as older male pigs begin to mature sexually (e.g., the testicles develop) and there is increased risk that the meat of these animals can express boar taint. If the pigs are left intact (i.e., with their testicles), approximately 10 to 20% of the meat will express boar taint. Most consumers perceive the taste and odor as very unpleasant. In Brazil, to ensure that meat is not contaminated by boar taint, all male pigs must be castrated prior to slaughter (Decree 9133 of 2017). The most commonly used technique in Brazil is surgical castration (removal of the testicles). Piglets are castrated between 3 and 10 days of age, usually by the farm staff. The use of medicines to relieve pain is not common in Brazil. An alternative to surgical castration is called immunocastration. The piglets receive two injections with a substance that restricts the development of the testicles. The injection does not contain hormones, but it causes the pig to produce antibodies against its own reproductive hormones. The risk of boar taint in the pigs that have been immunocastrated is eliminated. The method is approved and adopted in several countries, including Brazil.” **Sv2** “In Brazil, pigs raised for meat production are slaughtered at around 6 months of age. At that age, entire males (i.e., with the testicles) can express boar taint in the meat. Some consumers perceive this taste and smell as very unpleasant. Boar taint is associated with the production of male hormones. To ensure that meat does not have boar taint, male pigs used in meat production are castrated. Without testicles, the production of the hormones responsible for the boar taint in the meat does not occur. Another reason for castration is to reduce aggressive behavior between animals during rearing. Castration results in calmer animals. The most used castration technique in Brazilian commercial farms is surgical castration (removal of testicles). The piglets are castrated between 3 and 10 days of age. The use of medication to relieve pain during and after the procedure is not common. Another method used in Brazilian commercial farms is immunocastration. In this case, the piglets receive two injections (in weeks 8 and 16 of life) with a substance that restricts the development of the testicles. The injection does not contain hormones and leaves no residues in the meat. It works by causing the pig to produce antibodies against its own hormones, which inhibits the development of testicles and the boar taint in the meat of the pigs so treated. Lastly, it is possible to avoid castration and at the same time ensure that pigs do not develop the boar taint in the meat. To do this, entire (uncastrated) male pigs need to be slaughtered before the testicles develop and produce the hormones that lead to the boar taint. This may decrease the productivity of the farms a little, but eliminates the need for castration.”

## Appendix B

**Table A1:** Demographics of survey participants (Sv1, *n* = 441; Sv2, *n* = 768) and of the southern and general population, according to the latest Brazilian Institute of Geography and Statistics census (IBGE, 2011).

Variable	Sv1 Participants (%)	IBGE* (%)	Sv2 Participants (%)	IBGE* (%)
**Sex**				
Female	51	51	55	51
Male	49	49	45	49
**Age**				
18 to 24 years old	22	16	34	19
25 to 34 years old	29	23	19	24
35 to 44 years old	15	20	17	20
45 to 54 years old	17	18	16	16
55 years old and over	17	23	14	21
**Education**				
Up to high school	26	64	27	68
Undergraduate education (completed or ongoing)	74	36	73	32
**Current residence urban**	96	85	90	84
**Region of Brazil**				
South	100	100	24	15
Southwest			46	42
North			5	8
Northwest			15	28
Center-West			10	7
**Monthly family income**				
Up to 1 minimum wage			18	60
1 to 2 minimum wage			23	29
3 to 5 minimum wage			34	5
Over 5 minimum wage			25	5

## Appendix C

**Table A2:** Selected quotes extracted from the open-ended question, justifying attitudes towards different methods to deal with boar taint in pigs (Sv1, *n* = 441 southern Brazilian citizens; Sv2, *n* = 768 Brazilian citizens). Quotes are followed by participant number, gender (F = female, M = male), and participant’s treatment (the method to deal with boar taint in pigs). SC = surgical castration without pain control; SC+PC = surgical castration with pain control; IC = Immunocastration; EM = raising entire males.

Survey 1
*Pain and Suffering*
Causing unnecessary suffering to the animal is unacceptable, even more having effective methods for the same purpose (257, M, SC). I believe that castration is not the problem, but the pain that animals feel (161, M, SC). As a meat consumer I would be reassured knowing that the animal’s suffering has been minimized (376, F, SC+PC). Only with the use of medication to relieve pain, even so partially acceptable. The postoperative period is unlikely to be well done (186, M, SC). Minimization does not extinguish the animal’s pain, therefore, I consider this practice of castration unacceptable, even with the use of medications to minimize animal suffering, regardless of the associated costs (420, F, SC+PC).
*Ethical and Animal Welfare Concerns*
Nowadays, with so many techniques and the advancement of science, it is unacceptable that medieval procedures are still practiced! (258, M, SC). Every life is important, the animal supports us, the least to do is to treat it with respect and minimize its suffering (311, F, SC+PC). Animal welfare should be a priority, since we already use them as food. Their time in life should be as dignified and painless as possible (51, F, IC). I think that the food industry should seek new solutions to these issues mentioned above. Unfortunately, these issues are rarely considered and the financial aspects are valued at the expense of the animals’ quality of life (87, M, IC).
*Impact on Cost of Meat for Consumers*
Society must understand that the implementation of better treatment conditions for animals must be shared by all. If we are consumers, we are also responsible and we have to assume that (351, M, SC+PC). Regardless of the final cost, we should think about animals first, as it is cruel to castrate them like this. Those who want to consume meat must pay the price, because the animals cannot “pay the price’ (351, M, SC+PC). I think it is fair to increase the price to reduce animal suffering (416, M, SC+PC). I wouldn’t mind paying extra for something that will improve the quality of the meat and avoid pain in the animal (312, F, SC+PC). With the current economy, we do not want to pay more than we are used to, but considering the fact that the animal will not suffer pain, I believe we can pay more (49, F, IC). I would like to pay more to stop a practice that causes pain to animals. But I think it is important not to raise the price too much, to allow poor families to consume meat (13, F, CI). Priorities need to be weighed. Rising pork costs hurt the poorest (29, M, IC). Poor people have no income to support price increases (32, M, IC). If there is a big difference in price, it can make the product inaccessible to part of the population (52, F, IC). I believe that the welfare of the animal must be highly taken into account and I wouldn’t mind paying extra for it. However, I am aware that other individuals may not agree with this (overpaying) (309, F, SC+PC). From an economic point of view, pork is chosen as a favorable option, considering the cost of beef. By changing production costs, this amount will certainly be passed on to the consumer. I believe that it is up to the state to subsidize the costs of surgical castration to maintain the viability of production (356, M, SC+PC). Meat is already very expensive and the technique must be the responsibility of the government (71, F, IC).
*Effects on Production*
Knowing the socio-economic situation and the disparities between the regions, different ways of dealing with the issue are necessary in order to serve all producers and thus try to standardize production systems (219, M, SC). If there are conditions for the farmer he must do the ’chemical’ castration. But knowing the situation of many farmers, I believe that the cost of this castration would be very high for them (177, M, SC). Because there are small producers who probably do not have enough money for castration with vaccination (184, M, SC). I believe that surgical castration is cruel, because most of the time it is done without anesthesia, but I believe it is the most viable/practical/cheap method (234, F, SC).
*Naturalness*
Using a method like this leaves me in doubt as to whether it is really healthy, since it is not natural (118, F, IC). I imagine the physiological changes suffered by the animal given that it is a non-natural process of castration. Any non-normal action follows an abnormal reaction. Yes, for traditional castration (logically, done with hygiene and safety against pain and suffering of the animal) (133, M, IC). In my opinion, the production time of the pig should be natural, and not accelerated, reducing the time for fattening and slaughter in the shortest possible time as it is today. Regarding castration, it should be the most beneficial to the pig (399, F, SC+PC).
*Risks for Consumers (Residues in Meat)*
Until now, I did not know about chemical castration, I do not know the side effects (if any) in the pig when it grows, but if no side effects are proven, even for humans, I believe it would be the best alternative (199, M, SC). There should certainly be pain control. However, I do not know the costs/risks/benefits of immunocastration, so it is difficult to compare (194, F, SC). It does not seem healthy to animals and future meat consumers, as more substances would be introduced into the animals, and therefore more poisons that we eat (120, M, IM). I wonder about the spread of the “medicine” to the consumer’s table in immunocastration (233, F, SC). Even if the immunocastration does not contain hormones, will it really not come later to bring some ills to people? (390, SC+PC).
Survey 2
*Pain and Suffering*
It is the “least worst” of all other forms of castration, and I believe the animal feels the least pain (611, F, IC). I do not disapprove of castration, as long as it is painless for the animal (617, F, IC). Wrong. But castration is not wrong, what is wrong is not relieving pain (704, M, SC). It is not justifiable to castrate piglets without control of pain, but it is possible to castrate with anesthesia and post-surgical treatment, just as it is done for dogs and cats (903, F, SC). When they are young they will not feel much pain as they say (840, M, SC).
*Ethical and Animal Welfare Concerns*
The castration technique is very brutal, no matter how (80, M, EM). I think that pigs in Brazil are raised in a cruel and inhuman way. For me castration, trimming of the hooves and tail docking must be done with anesthesia to avoid pain in the animals (121, F, EM). I believe that measures that cause suffering to the animal will become increasingly unacceptable over time. Not adapting to the new demands can be a bad decision, since meat prepared in the laboratory and vegetables have been developing with speed (46, M, EM). It is unethical to cause pain to the animal. If there are other painless techniques, even if they increase costs or reduce production, these techniques MUST be used (784, M, SC). Castration is recommended in dogs. Why not pigs? (66, M, EM). If the cats I have at home are spayed, I cannot be against pigs’ castration (395, M, IC).
*Organoleptic Traits of Meat*
If it is done to avoid the odor in the meat, then the best thing to do is to castrate and solve the case (182, F, EM). If it leaves an odor in the meat, they must be castrated; however, painlessly (214, F, EM). I have no technical knowledge on the subject, but it seems to be a good way to remove the sexual odor from the meat (404, M, IC). I support castration because pain is inevitable in the animal, but it will be temporary and the product arrives at the consumer’s table with quality and no residual odors in the meat (887, M, SC). Surgical castration is an old practice and it (referring to raising entire males) can also cause repulsive meat (155, F, EM).
*Effects on Production*
According to the texts, I came to the conclusion that immunocastration appears to be the best alternative that causes the least possible stress to the animal while maintaining the production pace, appearing to be the most economically viable option and the easiest to accept in the pig market (563, F, IC). I think it is only acceptable if the cost of this procedure is much lower than those that do not cause pain because, in that case, the pain is relatively temporary (729, M, SC). While I disagree with the method, we need to consider small producers’ access to other castration techniques (731, M, SC). I support the method of injections to reduce the suffering of the animal and not decrease the productivity for the producer (since, being realistic, no producer will choose a practice that will decrease its production so much, unfortunately) (119, F, EM).
*Naturalness*
It would be a shorter course of the pig’s life, but more natural (41, F, EM). To maintain naturalness and the animal’s characteristic (52, M, EM). If it is the nature of the animal, I have no right to change it but it is just my opinion (68, F, EM). Castration is unnatural for any species (595, F, IC). For me, the ideal is for the animal to grow as natural as possible, a surgery without treatment of pain does not fit the ideal for me (667, M, SC).
*Risks for Consumers (Residues in Meat)*
I am against the introduction of substances in meat for consumption because I do not believe in their total elimination, and there is increasing accumulation in the consumer throughout his life (49, M, EM). I’m not sure about the residues from the hormone (390, M, IC). If it is with hormones, I don’t believe they will not stay in the meat afterwards (608, F, IC). We cannot keep modifying its nature to supply our selfishness, making the animal suffer and injecting medicines that go to meat and are absorbed by humans and that are not eliminated from our organism (184, F, EM). I support castration because pain is inevitable in animals, but it will be temporary, and the product will arrive at the consumer’s table with quality and without residual odors (887, M, SC).
