# Erratum: Hötzel, M.J.; et al. On the Road to End Pig Pain: Knowledge and Attitudes of Brazilian Citizens Regarding Castration. *Animals* 2020, *10*, 1826

**DOI:** 10.3390/ani11010138

**Published:** 2021-01-11

**Authors:** Maria José Hötzel, Maria Cristina Yunes, Bianca Vandresen, Rita Albernaz-Gonçalves, Raphaela E. Woodroffe

**Affiliations:** 1 Laboratório de Etologia Aplicada e Bem-Estar Animal, Universidade Federal de Santa Catarina, Rod. Admar Gonzaga 1346, Itacorubi, Florianópolis 88034-001, Brazil; mcyunes@hotmail.com (M.C.Y.); vandresen.bianca@gmail.com (B.V.); rita.albernaz@santarosa.ifc.edu.br (R.A.-G.); raphaela.w@hotmail.com (R.E.W.); 2 Instituto Federal Catarinense, Campus Santa Rosa do Sul, Santa Rosa do Sul 88965-000, Brazil

The authors wish to make the following corrections to this paper [1]:

Change in Acknowledgements:

We are grateful to the Brazilian Airport Infrastructure Company (INFRAERO) for allowing us to take Sv1 in the Airport of Florianópolis, and to the citizens that voluntarily participated in the study. The APC was funded by COST Action “Innovative approaches in pork production with entire males”-IPEMA (CA 15215), supported by COST (European Cooperation in Science and Technology).
