# Supplementary material for: On the Road to End Pig Pain: Knowledge and Attitudes of Brazilian Citizens Regarding Castration
Source: Animals (Basel). 2020 Oct 8;10(10):1826. doi: 10.3390/ani10101826 (PMC7650544; doi:10.3390/ani10101826)
Supplement: Supplementary file 1 [file animals-10-01826-s001.zip › Suplementary material_questionnaire.docx]

**Supplementary Table S1**: Script of the questionnaire survey 1 with the specific questions of the study and demographics

| Dear participant: This questionnaire is part of a survey from the University Federal in Santa Catarina about public opinion on farm animal production practices. Your participation is completely anonymous and voluntary. Only respondents 18 years old and over are invited to participate. You can opt out of participating at any time. |
| --- |
| 1. ( ) I agree to participate in this survey |
| PLEASE READ THE TEXT BELOW  *The majority of pigs in Brazil are slaughtered at around 6 months of age, as older male pigs begin to sexually mature (e.g. the testicles develop) and there is increased risk that the meat of these animals can express "boar taint" If the pigs are left intact (i.e. with their testicles), approximately 10 to 20% of the meat will express boar taint. Most consumers perceive the taste and odour as very unpleasant. In Brazil, to ensure that meat is not contaminated by boar taint, all male pigs must be castrated prior to slaughter (Decree 9133 of 2017).*  *The most commonly used technique in Brazil is surgical castration (removal of the testicles). Piglets are castrated between 3 and 10 days of age, usually by the farm staff. The use of medicines to relieve pain is not common in Brazil.*  *An alternative to surgical castration is called immunocastration. The piglets receive two injections with a substance that restricts the development of the testicles. The injection does not contain hormones, but it causes the pig to produce antibodies against its own reproductive hormones. The risk of boar taint in the pigs that have been immunocastrated is eliminated. The method is approved and adopted in several countries, including Brazil.* |
| Participants were randomly assigned to answer about one of the treatments: |
| 1. We would like to know your opinion on the subject discussed above   "It is acceptable to produce pork using the ‘surgical castration/ immunocastration’ technique described above " |
| Totally unacceptable (1) (2) (3) (4) (5) Totally acceptable |
| 1. Please justify the answers to the previous question:   Open question |
| 1. The statements below are all true. We want to know if you were aware of this information before answering this questionnaire   ( ) I knew ( ) I did not know |
| - Most pigs and poultry feeds used in Brazil are produced with transgenic soy and corn |
| - Surgical castration of pigs without pain control is the most common technique in Brazil |
| - Pig producers tail dock piglets to avoid them from being bitten by others in their group |
| - Meat from non-castrated pigs slaughtered after puberty may present boar taint |
| - In pig production in Brazil, antibiotics are commonly used to improve performance. Many of these antibiotics are the same as those used in human health |
| - Antibiotics used in animal production can contribute to the resistance of microorganisms to antibiotics |
| - Antibiotics fight only bacteria (not viruses and parasites) |
| - Several antibiotics used in animal production are also used in human health |
|  |
| **DEMOGRAPHIC QUESTIONS** |
| **Sex**  ( ) female  ( ) male |
| **Age**  ( ) 18-24 years old  ( ) 25-34 years old  ( ) 35-44 years old  ( ) 45-54 years old  ( ) 55-65 years old  ( ) over 65 years old |
| **Area where you live**  ( ) urban  ( ) rural |
| **Education**  ( ) up to high school  ( ) college or university (completed or ongoing) |
| **Do you have any kind of connection with animal production?**  ( ) not currently, but I grew up in an environment related to animal production  ( ) no |
| **How do you identify yourself in relation to your food consumption?**  ( ) I consume all products of animal origin  ( ) I'm a vegetarian  ( ) I'm vegan  ( ) I consume some types of animal products |
| **Mark your information sources on animal production**  ( ) television (rural programs)  ( ) internet  ( ) universities  ( ) products’ ads (meat, eggs or meat)  ( ) NGO campaigns |
| **How important is meat consumption to you?**  Not at all important (1) (2) (3) (4) (5) Very important |
| **How many days of the week do you consume meat (pork, beef, chicken, fish)?**  ( ) none  ( ) 1-2 days  ( ) 3-4 days  ( ) 5-7 days |
| **Please tick the reasons why you prefer pork in relation to other meat?**  ( ) organoleptic qualities like taste, texture, odour  ( ) price  ( ) tradition  ( ) ease of preparation  ( ) I don't eat pork |
| **Which source or sources of information on animal production do you trust?**  ( ) television (rural programs)  ( ) internet  ( ) universities  ( ) products’ ads (meat, eggs or meat)  ( ) NGO campaigns |
| **Have you or someone in your family or close friends experienced a medical condition involving antibiotic resistance?**  ( ) yes  ( ) no |

**Supplementary Table S2:** Script of the questionnaire survey 2 with the specific questions of the study and demographics.

| Dear participant: This questionnaire is part of a survey from the University Federal in Santa Catarina about public opinion on farm animal production practices. Your participation is completely anonymous and voluntary. Only respondents 18 years old and over are invited to participate. You can opt out of participating at any time by closing the survey before submitting it. |
| --- |
| 1. ( ) I agree to participate in this survey |
| **The ideal pig farm** |
| 1. Indicate the importance, in your personal opinion, of the elements below, in the context of production of meat for human consumption |
| \| *Mark only one oval per line. \| Avoiding husbandry practices that cause pain to animals. \| Avoiding husbandry practices that deprive animals of  freedom of movement. \| Ensuring that  meat is produced  free of  residues. \| \| --- \| --- \| --- \| --- \| \| More important \|  \|  \|  \| \| Less important \|  \|  \|  \| |
| 1. How do you identify yourself in relation to raising animals to produce food? |
| ( ) I am totally opposed to raising animals to produce food  (Respondent goes to question 12) |
| ( ) I support animal raising animals to produce food without restrictions |
| ( ) I support raising animals to produce food, as long as it is done ethically |
| PLEASE READ THE TEXTS BELOW TO ANSWER THE NEXT QUESTIONS |
| *In Brazil, pigs raised for meat production are slaughtered at around 6 months of age. At that age, entire males (i.e. with the testicles) can express boar taint in the meat. Some consumers perceive this taste and smell as very unpleasant. Boar taint is associated with the production of male hormones. To ensure that meat does not have boar taint, male pigs used in meat production are castrated. Without testicles, the production of the hormones responsible for the boar taint in the meat does not occur. Another reason for castration is to reduce aggressive behavior between animals during rearing. Castration results in calmer animals.*  *The most used castration technique in Brazilian commercial farms is surgical castration (removal of testicles). The piglets are castrated between 3 and 10 days of age. The use of medication to relieve pain during and after the procedure is not common.*  *Another method used in Brazilian commercial farms is immunocastration. In this case, the piglets receive two injections (in weeks 8 and 16 of life) with a substance that restricts the development of the testicles. The injection does not contain hormones and leaves no residues in the meat. It works by causing the pig to produce antibodies against its own hormones, which inhibits the development of testicles and the boar taint in the meat of the pigs so treated.*  *Lastly, it is possible to avoid castration and at the same time ensure that pigs do not develop the boar taint in the meat. To do this, entire (uncastrated) male pigs need to be slaughtered before the testicles develop and produce the hormones that lead to the boar taint. This may decrease the productivity of the farms a little, but eliminates the need for castration.* |
| 1. Did you read the text above? |
| ( ) Yes |
| Participants randomly assigned to answer about 1 of the treatments (surgical castration without pain relief/ immunocastration/ raising entire males) |
| **Your opinion about** (ONE OF THE 3 TREATMENTS) **to avoid boar taint in meat** |
| 1. Do you consider (ONE OF THE 3 TREATMENTS) adequate? |
| Totally inappropriate (1) (2) (3) (4) (5) Totally appropriate |
| 1. Do you approve of (ONE OF THE 3 TREATMENTS) ? |
| Totally disapprove (1) (2) (3) (4) (5) Totally approve |
| 1. Do you consider (ONE OF THE 3 TREATMENTS) acceptable? |
| Totally unacceptable (1) (2) (3) (4) (5) Totally acceptable |
| 1. Could you briefly justify your opinion on (ONE OF THE 3 TREATMENTS) without pain relief? |
| Open answer |
| **Your knowledge and opinion about pig production in Brazil** |
| The following questions are not a "test". They aim to assess knowledge and opinions of Brazilians on the subject. Answer based on the knowledge you had before participating in this questionnaire. |
| ( ) I knew ( ) I did not know |
| 1. Meat from non-castrated male pigs slaughtered after puberty may present boar taint. |
| 1. All male pigs used in meat production in Brazil undergo castration or immunocastration. |
| 1. Pig producers tail dock piglets to avoid them from being bitten by others in their group. This is done on the same day of castration and without the use of anaesthesia or pain relievers. |
| 1. In pig production in Brazil, antibiotics are commonly used to improve performance. Many of these antibiotics are the same as those used in human health. |
| 1. Most pigs and poultry feeds used in Brazil are produced with transgenic soy and corn. |
| **DEMOGRAPHIC QUESTIONS** |
| **Sex**  ( ) female  ( ) male |
| **Age**  ( ) 18-24 years old  ( ) 25-34 years old  ( ) 35-44 years old  ( ) 45-54 years old  ( ) 55-65 years old  ( ) over 65 years old |
| **Monthly family income**  ( ) I prefer not to say  ( ) up to 1 minimum wage  ( ) 1 to 2 minimum wages  ( ) 3 to 5 minimum wages  ( ) Over 5 minimum wages |
| **Region of residence in the country**  ( ) South  ( ) Southeast  ( ) North  ( ) Northeast  ( ) Centre-west |
| **Area where you live**  ( ) urban  ( ) rural |
| **Education**  ( ) up to high school  ( ) college or university (completed or ongoing) |
| **Do you have any kind of involvement with animal production?**  ( ) not currently, but I grew up in an environment related to animal production  ( ) no |
| **How do you identify yourself in relation to your food consumption?**  ( ) I consume all products of animal origin  ( ) I'm a vegetarian  ( ) I'm vegan  ( ) I consume some types of animal products |
| **How important is meat consumption to you?**  Not at all important (1) (2) (3) (4) (5) Very important |
| **How many days of the week do you consume meat (pork, beef, chicken, fish)?**  ( ) none  ( ) 1-2 days  ( ) 3-4 days  ( ) 5-7 days |
| **Do you consume pork?**  ( ) yes  ( ) no  ( ) rarely  ( ) sometimes |
| **Please tick the reasons why you prefer pork in relation to other meat?**  ( ) organoleptic qualities like taste, texture, odour  ( ) price  ( ) environmental impact  ( ) impact on animal welfare  ( ) tradition  ( ) ease of preparation |
| **Do you believe that the current context associated with COVID-19 may have influenced your responses?**  ( ) yes  ( ) no |
| **Do you have or ever had a castrated dog?**  ( ) yes  ( ) no |
